# Targeting the TL1A pathway in inflammatory bowel disease: mechanistic insights and emerging therapeutic pipeline

**DOI:** 10.1093/ibd/izag172

**Published:** 2026-08-27

**Authors:** Mohammed Nabil Quraishi, Vipul Jairath, Badr Al-Bawardy

**Affiliations:** Department of Gastroenterology, Sheikh Shakhbout Medical City, Abu Dhabi, United Arab Emirates; Institute of Cancer and Genomic Sciences, University of Birmingham, Birmingham, United Kingdom; College of Medicine and Health Sciences, Khalifa University of Science and Technology, Abu Dhabi, United Arab Emirates; Department of Medicine, Division of Gastroenterology, Western University, London, Ontario, Canada; Alimentiv, Inc, London, Ontario, Canada; London Health Research Institute, London, Ontario, Canada; Department of Epidemiology and Biostatistics, University of Western Ontario, London, Ontario, Canada; Department of Gastroenterology, King Faisal Specialist Hospital and Research Center, Riyadh, Saudi Arabia; Department of Internal Medicine, Section of Digestive Diseases, Yale School of Medicine, New Haven, CT, United States; College of Medicine, Alfaisal University, Riyadh, Saudi Arabia

**Keywords:** TNFSF15, intestinal fibrosis, biologic therapy, precision medicine

## Abstract

Tumor necrosis factor-like ligand 1A (TL1A), encoded by TNFSF15, signals through death receptor 3 on effector T cells, innate lymphoid cells, and intestinal myofibroblasts, driving both chronic intestinal inflammation and tissue fibrosis. In this narrative review, we provide a contemporary appraisal of TL1A-directed therapeutics in inflammatory bowel disease based on electronic searches through May 2026. Three anti-TL1A monoclonal antibodies (tulisokibart, afimkibart, and duvakitug) have advanced to phase 3 trials across multiple global programs, with phase 2 clinical remission rates of 26%-48% in ulcerative colitis (placebo-adjusted differences 15-26 percentage points) and endoscopic response rates of 26%-48% in Crohn’s disease, alongside favorable safety profiles. Mucosal transcriptomic and serum proteomic analyses from phase 2 trials, including DDW 2026 readouts from ARTEMIS-UC and TUSCANY-2, provide the first human-tissue confirmation of class antifibrotic activity through the suppression of Th17, myeloid, and extracellular matrix pathways. TNFSF15 risk-variant companion diagnostics are advancing, although their incremental utility remains modest and will be definitively tested in phase 3. The pipeline has expanded to at least 19 development programs, including extended half-life antibodies (XmAb942, SPY002, and BCD-261), bispecifics combining TL1A with IL-23 or α4β7 (RO7837195, XmAb412, LQ080, and ALX001), a first-in-class oral anti-TL1A nanobody, and a first-in-class DR3 receptor antagonist (SL-325). We discuss therapeutic positioning and timing, the opportunity in acute severe UC, and the unusual standing of IBD as the lead indication for this mechanism class. Phase 3 results anticipated in 2026-2027 will be particularly informative for fibrostenotic phenotypes and biologic-refractory patients.

Key messages *What is already known?*

- TL1A–DR3 signaling drives intestinal inflammation and fibrosis; three anti-TL1A antibodies have completed phase 2 with consistent UC and CD efficacy.

*What is new here?*

- We integrate phase 3 design analytics, antifibrotic clinical findings, an expanded pipeline (oral nanobodies, bispecifics, and DR3 antagonism), and analyses of therapeutic timing and IBD-as-lead-indication.

*How can this study help patient care?*

- Clinicians can anticipate where TL1A inhibitors will sit in the treatment algorithm by phenotype and prior-therapy exposure as phase 3 readouts emerge over 2026-2027.

## Introduction

Inflammatory bowel disease (IBD), encompassing Crohn’s disease (CD) and ulcerative colitis (UC), affects approximately 7 million individuals worldwide, with rising incidence in newly industrialized regions.^1^^,^^2^ Despite significant therapeutic advances over the past two decades, including tumor necrosis factor (TNF) inhibitors, integrin antagonists, interleukin (IL)-12/23 blockers, Janus kinase (JAK) inhibitors, sphingosine 1-phosphate receptor modulators (S1PRM), and selective IL-23 inhibitors, a substantial proportion of patients fail to achieve or maintain remission. The “therapeutic ceiling” for advanced therapies in IBD, characterized by placebo-adjusted remission rates of 10%-25%, represents a persistent challenge in clinical practice.^3–5^

Beyond inflammation, the development of intestinal fibrosis poses a particularly intractable problem. Up to 50% of patients with CD develop stricturing complications within 20 years of diagnosis, frequently requiring surgical intervention despite immunosuppressive therapy.^6^ Similarly, chronic UC is increasingly recognized to be associated with submucosal fibrosis that may contribute to treatment refractoriness and functional impairment.^7^^,^^8^ Current biologics and small molecules primarily target inflammatory pathways but lack demonstrated antifibrotic efficacy, leaving a critical therapeutic gap.

Against this backdrop, tumor necrosis factor-like ligand 1A (TL1A), encoded by the *TNFSF15* gene, has emerged as a compelling therapeutic target with the potential to address both inflammatory and fibrotic disease mechanisms.^9^^,^^10^ First identified in 2002 as a novel TNF superfamily member, TL1A has accumulated over two decades of genetic, immunological, and translational evidence supporting its central role in IBD pathogenesis (Figure 1).^11^^,^^12^ The pathway represents the first genuinely novel mechanism of action to reach phase 3 development for IBD since the advent of anti-integrin therapy, marking a potential inflection point in the therapeutic landscape.

**Figure 1:**
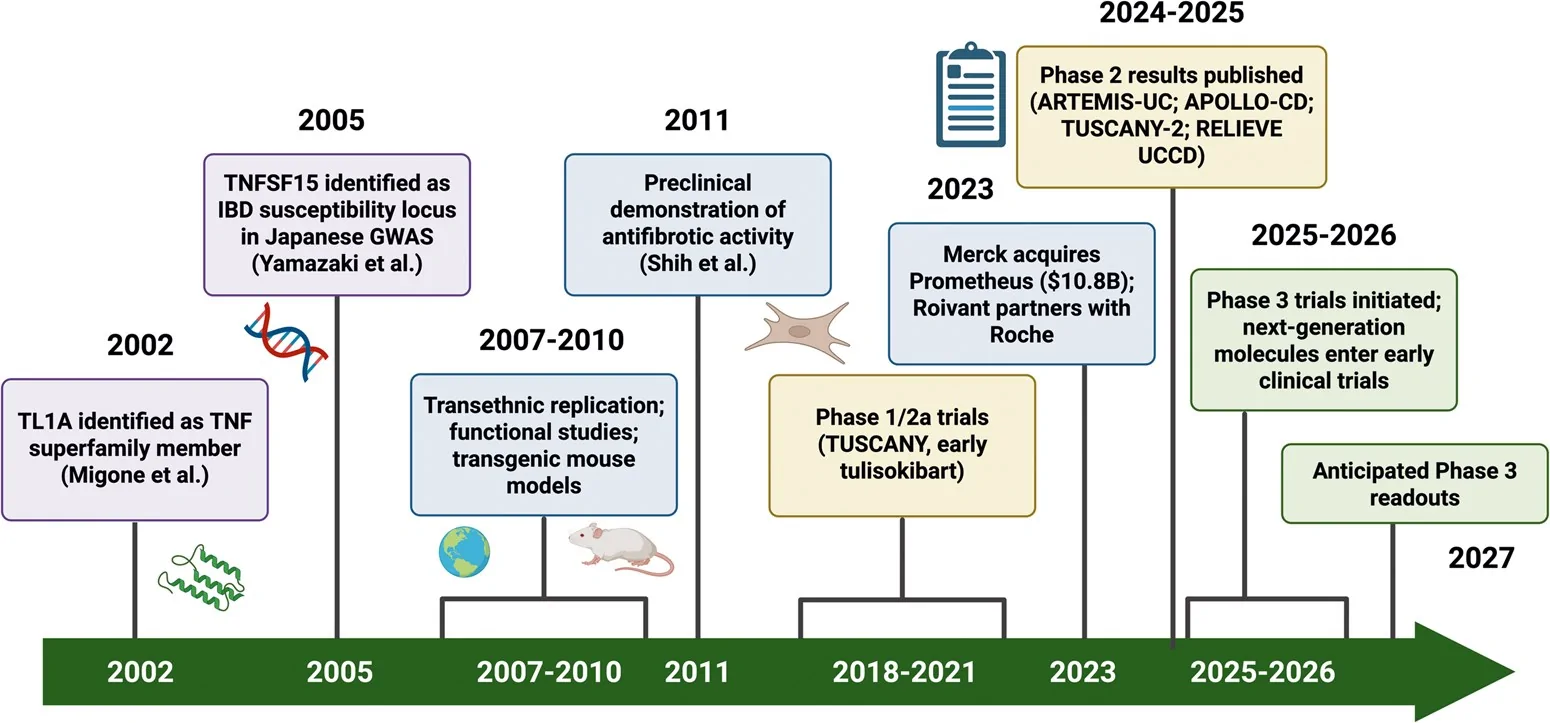
Key milestones in TL1A research and therapeutic development. Timeline illustrating the translational journey of TL1A from target identification to phase 3 clinical trials. 2002: TL1A identified as a TNF superfamily member (Migone et al.). 2005: TNFSF15 identified as an IBD susceptibility locus in Japanese GWAS (Yamazaki et al.). 2007-2010: Transethnic replication studies, functional characterization, and transgenic mouse models established. 2011: Preclinical demonstration of antifibrotic activity (Shih et al.). 2018-2021: phase 1/2a trials initiated (TUSCANY, early tulisokibart studies). 2023: Major commercial validation with MSD’s acquisition of Prometheus for U.S.$10.8 billion; Roivant partners with Roche. 2024-2025: phase 2 results published from ARTEMIS-UC, APOLLO-CD, TUSCANY-2, and RELIEVE UCCD trial. 2025-2026: phase 3 registrational trials initiated; next-generation molecules (extended half-life antibodies, bispecifics) enter early clinical development. 2027: Anticipated phase 3 readouts. Abbreviations: GWAS, genome-wide association study; IBD, inflammatory bowel disease; TL1A, TNF-like ligand 1A; TNFSF15, tumor necrosis factor superfamily member 15.

Three anti-TL1A monoclonal antibodies—tulisokibart (MK-7240), afimkibart (RO7790121), and duvakitug (TEV-48574)—have demonstrated clinical efficacy in phase 2 trials and have advanced to phase 3 programs.^13–17^ Concurrently, a pipeline of over 16 additional programs, including extended half-life antibodies, bispecific constructs, and a first-in-class DR3 receptor antagonist, signals sustained pharmaceutical investment exceeding U.S.$20 billion in acquisitions and licensing since 2023.^18^ This review provides a comprehensive synthesis of TL1A biology, the genetic rationale for therapeutic targeting, the mechanistic basis for dual anti-inflammatory and antifibrotic activity, clinical trial evidence, and the rapidly evolving pipeline. We critically appraise the positioning of TL1A inhibitors within the current IBD treatment algorithm and consider future directions, including precision medicine approaches and combination strategies.

### Approach to the literature

An electronic search was conducted in MEDLINE, Embase, and the Cochrane Library up to 16th of May 2026 using terms related to TL1A/TNFSF15, IBD, and specific therapeutic agents (Figure S1—PRISMA Flow Diagram with detailed search strategy). Reference lists of selected articles, systematic reviews, and conference abstracts from major gastroenterology meetings (DDW, ECCO, UEG Week, and ACG) and clinical trial registries (ClinicalTrials.gov, EudraCT) were also reviewed. Articles in any language were considered; duplicate publications were resolved by retaining the most recent study. Given the heterogeneity of available data, a narrative review was conducted rather than formal meta-analysis. A formal risk-of-bias assessment was not performed. Data available only in conference abstracts or posters, including presentations at DDW 2026 and ECCO 2026, should be regarded as preliminary pending full peer-reviewed publication. The duvakitug RELIEVE UCCD trials were published in full in July 2026, after this cutoff.

## TL1A: discovery, structure, and expression

### Identification and molecular characterization

TL1A was identified in 2002 by Migone et al.^11^ as a type II transmembrane protein belonging to the TNF superfamily (TNFSF15). The molecule functions as a T-cell co-stimulator, with structural homology to TNF and binding affinity for death domain receptor 3 (DR3, also known as TNFRSF25), which exhibits the highest homology to TNF receptor 1 (TNFR1) among TNFRSF members.^19^^,^^20^ TL1A exists in both membrane-bound and soluble forms following proteolytic cleavage; both forms retain biological activity and contribute to diverse immunological effects.^21^

Structural studies have elucidated the biochemical properties of the human TL1A ectodomain, revealing a typical TNF homology domain that enables homotrimerization and receptor engagement.^22^ This trimeric assembly is essential for functional signaling, with receptor clustering triggering downstream activation of NF-κB and MAPK pathways.^23^ In addition to DR3, TL1A can bind decoy receptor 3 (DcR3/TNFRSF6B), which exists exclusively in soluble form and serves as a competitive inhibitor of TL1A signaling. Notably, DcR3 is absent from mouse and rat genomes, complicating the direct translation of preclinical findings to human disease.^24^

### Cellular sources and regulation

TL1A expression is predominantly induced rather than constitutive, with primary sources including activated antigen-presenting cells (dendritic cells, macrophages, and monocytes), T lymphocytes, and endothelial cells.^25^^,^^26^ Microbial stimulation through Toll-like receptors (TLRs) and Fcγ receptor engagement on myeloid cells potently upregulates TL1A expression, establishing a mechanistic link between mucosal microbial sensing and adaptive immune activation.^27^ The microbiota-inducible nature of TL1A is particularly relevant to IBD, where dysbiosis and impaired barrier function permit increased bacterial translocation. DR3 expression is more restricted, being largely confined to lymphocytes (particularly T cells), innate lymphoid cells (ILCs), and more recently recognized populations, including intestinal fibroblasts.^28^^,^^29^ This expression pattern positions the TL1A–DR3 axis at the interface of innate and adaptive immunity, with the capacity to influence both immediate inflammatory responses and longer-term tissue remodeling processes.

### Expression in IBD

Both TL1A and DR3 are markedly upregulated in inflamed intestinal tissues from patients with IBD relative to healthy mucosa.^30^^,^^31^ Immunohistochemical and transcriptomic analyses demonstrate elevated TL1A in lamina propria mononuclear cells and epithelial compartments, with parallel increases in soluble TL1A detectable in serum and intestinal fluid during active disease.^32^ Importantly, elevated TL1A levels correlate with disease activity and decline in response to effective biological therapy, supporting the pathway’s pathophysiological relevance and potential utility as a biomarker.^33^

## Mechanistic basis for therapeutic targeting

### TL1A–DR3 signaling in intestinal inflammation

Engagement of DR3 by TL1A activates multiple downstream signaling cascades, including NF-κB, MAPK (ERK, JNK, and p38), and PI3K/Akt pathways.^23^^,^^34^ These signals promote T-cell activation, proliferation, and cytokine production, synergizing with T-cell receptor engagement to amplify Th1, Th2, and Th17 responses and drive production of IFN-γ, IL-17, and TNF (Figure 2A).^34^^,^^35^ Beyond these canonical effector populations, Th9 cells have been identified as a TL1A/DR3-regulated effector population in CD; DR3 signaling promotes Th9 differentiation and their proinflammatory phenotype, with DR3-expressing Th9 cells driving severe intestinal inflammation upon adoptive transfer while an anti-IL-9 antibody ameliorates established ileitis.^36^ Th9-related gene signatures were similarly upregulated in CD mucosal biopsies, suggesting that suppression of pathogenic Th9 responses may contribute to TL1A inhibitor efficacy.^37^

**Figure 2:**
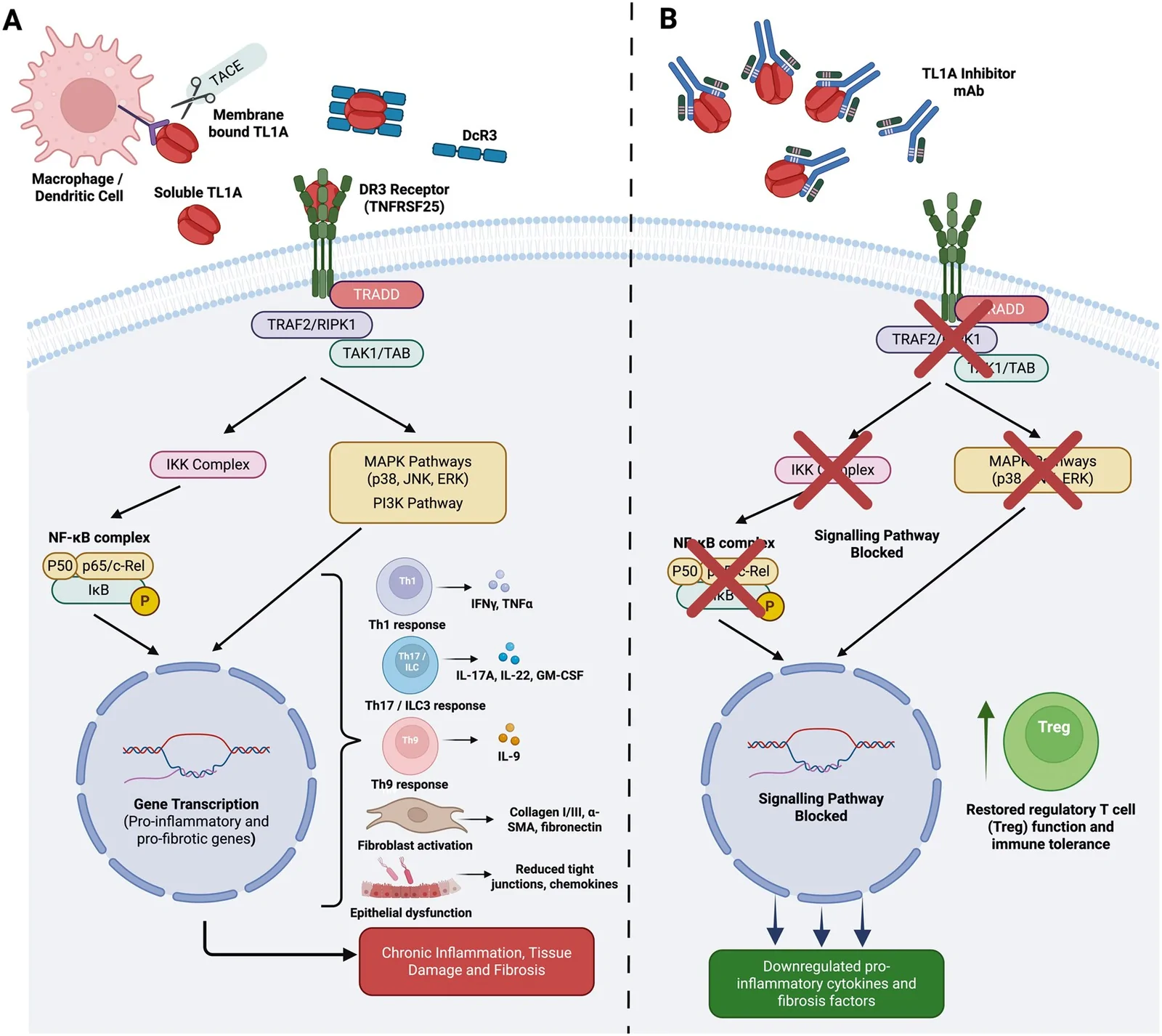
TL1A–DR3 signaling pathway and effects of therapeutic inhibition. (A) Pathogenic TL1A signaling: TL1A, produced by macrophages and dendritic cells as membrane-bound or soluble (cleaved by TACE) forms, binds to DR3 (TNFRSF25) on target cells. DcR3 serves as a soluble decoy receptor. DR3 engagement activates intracellular signaling through TRADD, TRAF2/RIPK1, and TAK1/TAB, leading to NF-κB activation and MAPK/PI3K pathway stimulation. This drives Th1, Th17/ILC3, and Th9 responses with the production of pro-inflammatory cytokines (IFN-γ, TNF-α, IL-17A, IL-22, GM-CSF, and IL-9), fibroblast activation with extracellular matrix production (collagen I/III, α-SMA, fibronectin), and epithelial dysfunction, culminating in chronic inflammation, tissue damage, and fibrosis. (B) TL1A inhibition: Monoclonal antibody blockade of TL1A prevents DR3 engagement, blocking downstream NF-κB and MAPK signaling pathways. This results in downregulated pro-inflammatory cytokines and fibrosis factors and restored regulatory T cell (Treg) function and immune tolerance. Abbreviations: α-SMA, alpha-smooth muscle actin; DcR3, decoy receptor 3; DR3, death receptor 3; ERK, extracellular signal-regulated kinase; GM-CSF, granulocyte-macrophage colony-stimulating factor; IFN-γ, interferon-gamma; IκB, inhibitor of kappa B; IL, interleukin; ILC3, type 3 innate lymphoid cell; JNK, c-Jun N-terminal kinase; mAb, monoclonal antibody; MAPK, mitogen-activated protein kinase; NF-κB, nuclear factor kappa B; PI3K, phosphoinositide 3-kinase; RIPK1, receptor-interacting protein kinase 1; TAB, TAK1-binding protein; TACE, TNF-α converting enzyme; TAK1, transforming growth factor-β-activated kinase 1; Th1/Th9/Th17, T helper type 1/9/17 cells; TL1A, TNF-like ligand 1A; TNF-α, tumor necrosis factor-alpha; TNFRSF25, TNF receptor superfamily member 25; TRADD, TNFR1-associated death domain protein; TRAF2, TNF receptor-associated factor 2; Treg, regulatory T cell.

Outside conventional T-helper subsets, TL1A–DR3 signaling exerts critical effects on innate lymphoid cells. ILC2s, which express high levels of DR3, respond to TL1A with enhanced IL-13 production, driving type 2 inflammatory responses in the small intestine.^38^ ILC3s similarly express DR3 and respond to TL1A with increased IL-22 production and proliferation, potentially influencing mucosal barrier function and epithelial regeneration.^39^ This capacity to orchestrate both type 1/17 and type 2 immune responses distinguishes TL1A from more narrowly targeted cytokine pathways. TL1A–DR3 signaling also intersects with regulatory T-cell biology. DR3 is constitutively and highly expressed on FoxP3-positive regulatory T cells, and DR3 co-stimulation can selectively expand functional, suppressive regulatory T cells; however, sustained TL1A exposure also accelerates their turnover while attenuating suppressive capacity, so the net effect on mucosal tolerance is context-dependent.^40^^,^^41^

Evidence from transgenic mouse models provides compelling support for TL1A’s causal role in intestinal inflammation. Mice with constitutive TL1A overexpression in myeloid or lymphoid compartments develop spontaneous small intestinal inflammation resembling CD, with expansion of Th1 and Th2 effector populations.^42^^,^^43^ Conversely, genetic deletion of *Tnfrsf25* (DR3) ameliorates inflammation across several colitis models, including dextran sodium sulfate (DSS)-induced and T-cell transfer colitis, and in Crohn’s-like ileitis DR3 deletion normalizes mucosal inflammatory gene expression and prevents disease. Ligand and receptor are not equivalent, however: TL1A (*Tnfsf15*) is dispensable for the development of ileitis and influences only the kinetics of inflammation, indicating that the pro-inflammatory role of DR3 is only partially mediated by TL1A and raising the possibility of additional DR3 ligands.^44^^,^^45^

### Antifibrotic potential

Perhaps the most distinctive feature of TL1A biology is its involvement in tissue fibrosis, a disease complication for which no approved therapy exists in IBD. Intestinal fibrosis remains a major clinical challenge in CD, where stricturing disease frequently necessitates surgical resection despite immunosuppressive treatment.^46^ TL1A–DR3 signaling has emerged as a direct mediator of profibrotic processes independent of its inflammatory effects (Figure 3). Mechanistically, TL1A promotes fibrogenesis through multiple pathways. TL1A induces myofibroblast activation and collagen synthesis via the TGF-β1/Smad3 pathway, with contribution from Rho kinase signaling.^47–49^ Additionally, TL1A promotes epithelial-mesenchymal transition (EMT), with colonic specimens from IBD patients demonstrating an inverse correlation between TL1A levels and the epithelial marker E-cadherin, alongside positive correlations with mesenchymal markers FSP1 and α-smooth muscle actin (α-SMA).^47^

**Figure 3:**
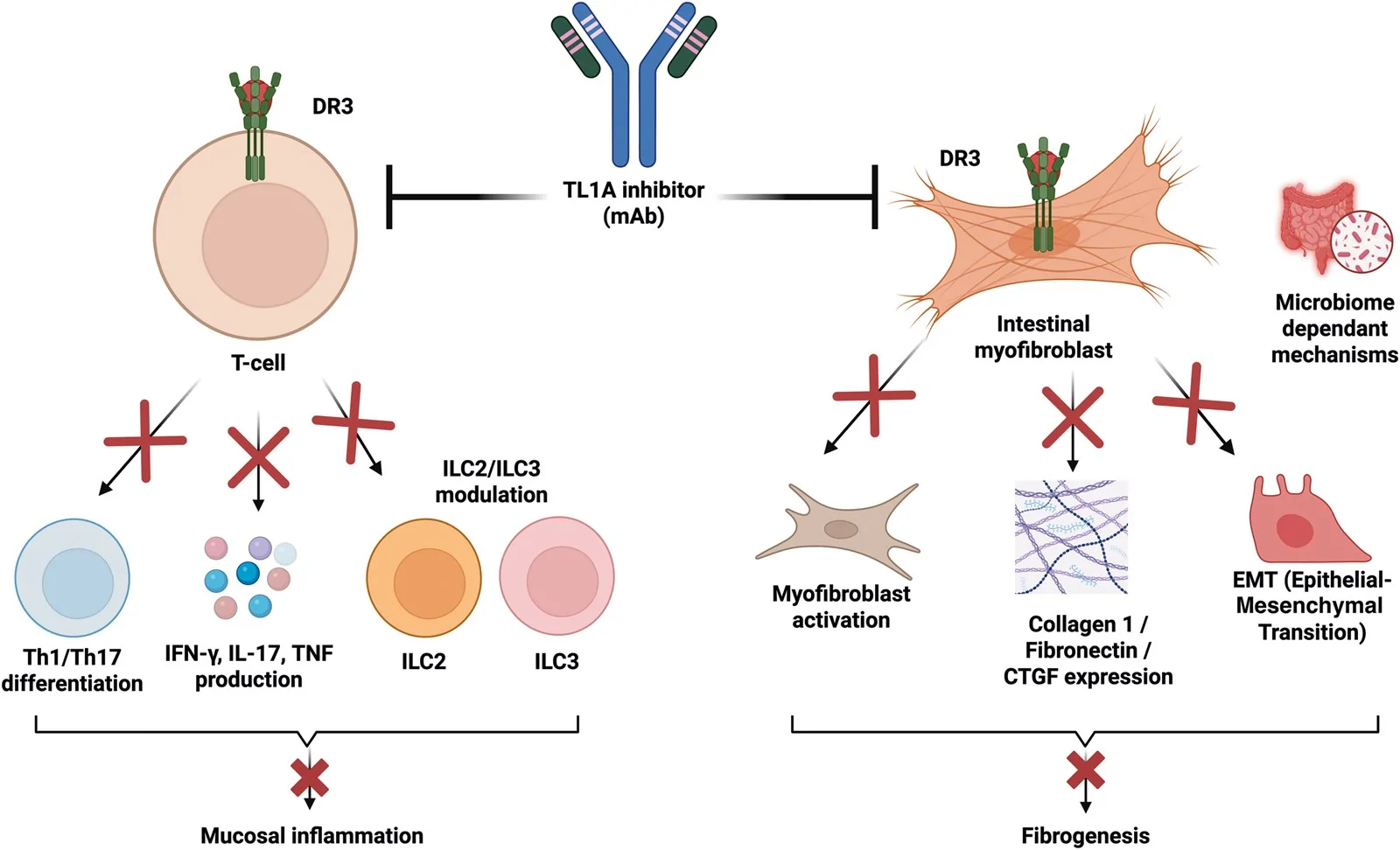
Dual anti-inflammatory and antifibrotic mechanism of TL1A inhibition. TL1A inhibitors (monoclonal antibodies) block the TL1A–DR3 interaction on both immune cells and intestinal myofibroblasts. Left panel (anti-inflammatory effects): TL1A blockade on T cells inhibits Th1/Th17 differentiation, reduces pro-inflammatory cytokine production (IFN-γ, IL-17, TNF), and modulates ILC2/ILC3 activity, collectively reducing mucosal inflammation. Right panel (antifibrotic effects): TL1A blockade on intestinal myofibroblasts inhibits myofibroblast activation, reduces extracellular matrix deposition (collagen I, fibronectin, CTGF), and prevents epithelial-mesenchymal transition (EMT), thereby reducing fibrogenesis. Microbiome-dependent mechanisms may also contribute to antifibrotic effects. This dual mechanism distinguishes TL1A inhibitors from existing IBD biologics and suggests potential efficacy in fibrostenotic disease. Abbreviations: CTGF, connective tissue growth factor; DR3, death receptor 3; EMT, epithelial-mesenchymal transition; IFN-γ, interferon-gamma; IL-17, interleukin-17; ILC2, type 2 innate lymphoid cell; ILC3, type 3 innate lymphoid cell; mAb, monoclonal antibody; Th1, T helper type 1 cell; Th17, T helper type 17 cell; TL1A, TNF-like ligand 1A; TNF, tumor necrosis factor.

A particularly intriguing aspect of TL1A-mediated fibrosis is its dependence on the gut microbiome. In TL1A-transgenic mice, intestinal fibrosis develops only under conventional housing and is absent in germ-free conditions.^48^ Specific microbial taxa, including Helicobacter and segmented filamentous bacteria, appear to amplify TL1A-driven fibrogenesis. Conversely, broad-spectrum antibiotic depletion attenuates fibrosis even in established disease.^12^ These findings suggest that TL1A integrates microbial cues with stromal activation and that TL1A blockade may decouple inflammation from microbiota-driven remodeling—a feature with potential relevance to the clinical heterogeneity of fibrostenotic Crohn’s disease.

The profibrotic role of TL1A extends beyond the intestine. In mouse models of airway inflammation, neutralization of TL1A or genetic deletion of DR3 restricted peribronchial smooth muscle hypertrophy and lung collagen accumulation, with human lung fibroblasts and bronchial epithelial cells expressing DR3 and responding to TL1A by proliferating and producing collagen and periostin.^50^ This tissue-agnostic mechanism supports the ongoing investigation of TL1A inhibitors in systemic sclerosis-associated interstitial lung disease (SSc-ILD; ATHENA-SSc-ILD, NCT05270668).

### Translational biomarker evidence

First-in-human studies have provided direct evidence that TL1A inhibition reverses both inflammatory and fibrotic gene signatures in diseased tissue. In the phase 2a TUSCANY trial, colonic biopsies from UC patients treated with afimkibart demonstrated reduced expression of genes associated with extracellular matrix remodeling and fibrosis alongside suppression of inflammatory transcripts.^51^ Two independent class-level datasets (presented as a conference abstract) have extended these findings: bulk mucosal-biopsy transcriptomics from ARTEMIS-UC showed sustained suppression of fibrosis-related, Th1 and Th17 gene signatures with tulisokibart through week 50, and high-sensitivity serum proteomics from TUSCANY-2 (*n* = 135) demonstrated normalization of OSM, AREG, HGF, MMP8, and MMP12 toward healthy-control levels with afimkibart, indicating that anti-TL1A blockade modulates stromal and tissue-remodeling biology in patients in vivo, not only in preclinical models.^52^^,^^53^ These translational biomarker data support the mechanistic hypothesis that TL1A blockade may address both the inflammatory and structural consequences of chronic IBD (Figure 2B). These findings should nonetheless be interpreted with caution. The available human evidence for an antifibrotic effect derives from mucosal transcriptomic, serum proteomic, and biomarker analyses rather than from direct demonstration of fibrosis regression, and modulation of extracellular matrix signatures, while mechanistically encouraging, does not by itself establish clinical antifibrotic efficacy or reversal of established fibrostenotic disease.

## Clinical development of TL1A inhibitors

Three anti-TL1A monoclonal antibodies have completed phase 2 development and advanced to phase 3 registrational trials, each demonstrating clinically meaningful efficacy in both UC and CD (Tables 1 and 2). Beyond these first-generation agents, an expanding pipeline of next-generation molecules, including extended half-life antibodies, bispecifics, and DR3 antagonists, reflects sustained pharmaceutical investment in this therapeutic class (Figure 4).

**Figure 4:**
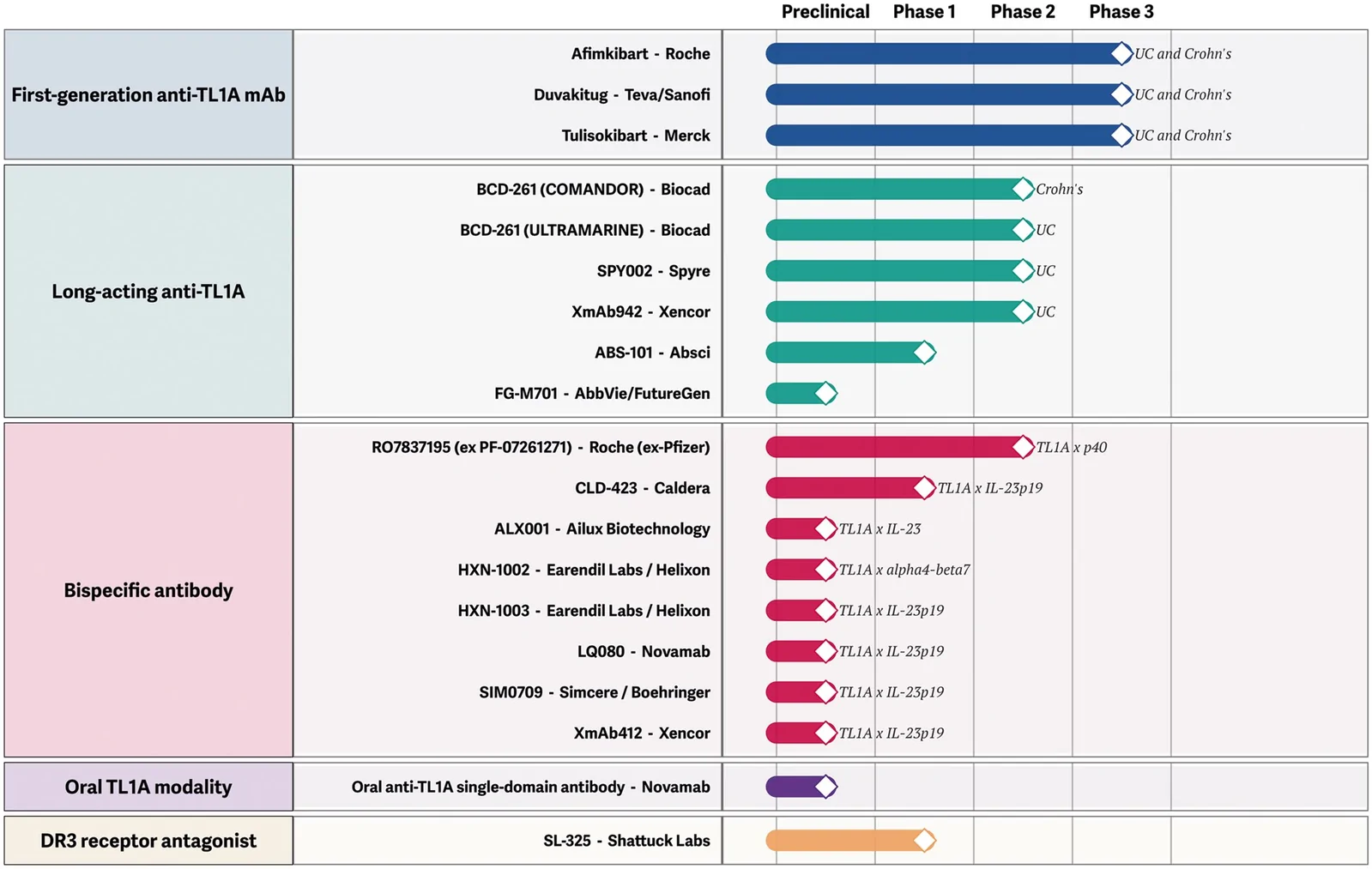
Clinical development pipeline for TL1A-targeted therapeutics in IBD. Nineteen development programs for IBD are organized by therapeutic modality and arranged horizontally by current development stage (preclinical, phase 1, phase 2, and phase 3) as of May 2026. Endpoint markers indicate the most advanced ongoing program for each molecule. Indication (UC, Crohn’s, or UC and Crohn’s) is shown to the right of each entry; for bispecific antibodies, the target combination is shown instead. First-generation anti-TL1A monoclonal antibodies (tulisokibart, afimkibart, and duvakitug) are in phase 3 registrational trials in both UC and Crohn’s disease. Long-acting anti-TL1A antibodies engineered for extended dosing intervals are advancing through phase 2 (XmAb942 in UC; BCD-261 in two parallel trials, COMANDOR in Crohn’s disease and ULTRAMARINE in UC; SPY002 in UC within the SKYLINE-UC platform), phase 1 (ABS-101), and preclinical development (FG-M701). Bispecific antibodies combining TL1A with a second cytokine or integrin target represent the most active preclinical category and include RO7837195 (TL1A × p40, the most clinically advanced bispecific at phase 2b), CLD-423 (phase 1, TL1A × IL-23p19), and several preclinical candidates (XmAb412, HXN-1002, HXN-1003, SIM0709, LQ080, ALX001). A first-in-class oral anti-TL1A single-domain antibody (Novamab) and SL-325 (Shattuck Labs), the first clinical-stage DR3 receptor antagonist, complete the pipeline. Abbreviations: alpha4-beta7, alpha-4 beta-7 integrin; CD, Crohn’s disease; DR3, death receptor 3; IL, interleukin; mAb, monoclonal antibody; SC, subcutaneous; TL1A, TNF-like ligand 1A; UC, ulcerative colitis.

**Table 1:** Phase 2 clinical trial designs and efficacy results for TL1A inhibitors in IBD.

Feature	Tulisokibart (ARTEMIS-UC)	Tulisokibart (APOLLO-CD)	Afimkibart (TUSCANY-2)	Duvakitug (RELIEVE UCCD)
**Trial design**				
**NCT number**	NCT04996797	NCT05013905	NCT04090411	NCT05499130
**Phase**	2a	2a	2b	2b
**Disease**	UC	CD	UC	UC and CD (basket)
**Design**	RCT, double-blind, placebo-controlled	Open-label, single-arm	RCT, double-blind, treat-through	RCT, double-blind, basket trial
**Comparator**	Placebo	Historical placebo	Placebo	Placebo
**N enrolled**	135 (C1) + 43 (C2)	55	246	137 (UC) + 138 (CD)
**Randomization**	1:1	N/A	9-arm adaptive (placebo-to-active and active-to-active dose combinations)	1:1:1
**Precision medicine**	Genetic CDx (C1: all; C2: BM + only)	Genetic CDx tested	Genetic biomarker (prespecified)	Stratified by prior AT
**Induction regimen**				
**Route**	IV	IV	SC	SC
**Loading dose**	1000 mg (day 1)	1000 mg (day 1)	None	2250 mg (day 1)
**Induction doses**	500 mg (wks 2, 6, 10)	500 mg (wks 2, 6, 10)	50, 150, or 450 mg	450 or 900 mg
**Frequency**	Q4W	Q4W	Q4W	Q2W
**Duration**	12 weeks	12 weeks	12 weeks	14 weeks
**Maintenance design**				
**Design type**	Responder re-randomization (OLE)	N/A	Treat-through	LTE for responders
**Maintenance doses**	100 vs 250 mg SC	N/A	Same as induction	Q4W
**Duration**	To week 50	N/A	40 weeks (to week 56)	To week 24+
**Statistical approach**				
**Analysis method**	Frequentist; ranked endpoints; NRI; *P* < .05	Historical comparison; *P* < .05	Frequentist (Chan-Zhang); NRI; *P* < .05	Bayesian; >90% posterior probability
**Primary efficacy results**				
**Primary endpoint**	Clinical remission (MMS)	Endoscopic response (≥50% SES-CD↓)	Clinical remission (tMS)	UC: remission (MMS); CD: endo response
**Active arm (%)**	26%	26%	26%, 23%, 24% (by dose)	UC: 36%, 48%; CD: 26%, 48%
**Placebo (%)**	1%	12%^a^	12%	UC: 20%; CD: 13%
**Δ vs placebo (pp)**	25	14	14, 11, 12	UC: 15, 26; CD: 13, 33
**Significance**	*P* < .001	*P* = .002	NS (tMS); sig (mMS)	>90% posterior
**BM + enrichment**	32% vs 11% (21 pp Δ)	Alt algorithm: 57%	Enhanced in BM+	Consistent across subgroups

Abbreviations: AT, advanced therapy; BM+, biomarker-positive; C1/C2, cohort 1/2; CD, Crohn’s disease; CDx, companion diagnostic; endo, endoscopic; IV, intravenous; LTE, long-term extension; MMS, modified Mayo score; NRI, nonresponder imputation; NS, not significant; OLE, open-label extension; pp, percentage points; Q2W, every 2 weeks; Q4W, every 4 weeks; RCT, randomized controlled trial; SC, subcutaneous; SES-CD, Simple Endoscopic Score for Crohn’s Disease; sig, significant; tMS, total Mayo score; UC, ulcerative colitis.

a Historical placebo estimate.

**Table 2:** Phase 3 clinical program designs for TL1A inhibitors in IBD.

Feature	Tulisokibart (MSD)	Afimkibart (Roche)	Duvakitug (Teva/Sanofi)
**Program overview**			
** UC induction trials**	ATLAS-UC (MK-7240-001) (NCT06052059) Study 1 + Study 2	AMETRINE-1 (NCT06589986) AMETRINE-2 (NCT06588855)	SUNSCAPE-1 (NCT07184996)
**CD induction trials**	ARES-CD (MK-7240-008) (NCT06430801) Study 1 + Study 2	SIBERITE-1 (NCT06819878) SIBERITE-2 (NCT06819891)	STARSCAPE-1 (NCT07184931)
**Maintenance trials**	Integrated within induction protocols	Integrated within AMETRINE-1 and SIBERITE-1	SUNSCAPE-2 (NCT07185009) STARSCAPE-2 (NCT07184944)
**Extension study**	NCT06651281 (combined UC/CD)	Planned	OLE integrated within maintenance studies
**Trial design**			
**Overall design**	RCT, double-blind, placebo-controlled	RCT, double-blind, placebo-controlled, treat-through	RCT, double-blind, placebo-controlled; separate induction and maintenance studies
**Maintenance approach**	Integrated induction-maintenance within single protocol	Treat-through (all randomized patients continue to maintenance)	Responder re-randomization (clinical responders enter separate maintenance study)
**Statistical approach**	Frequentist	Frequentist	Frequentist (changed from Bayesian in phase 2)
**Route of administration**			
**Induction**	IV induction	IV induction (SC formulation in phase 2)	SC induction
**Maintenance**	SC maintenance	SC maintenance	SC maintenance
**Primary endpoints**			
**UC induction**	Clinical remission per mMS (Week 12)	Clinical remission per mMS (Week 12)	Clinical remission per mMS (Week 12)
**UC maintenance**	Clinical remission per mMS (Week 52)	Clinical remission per mMS (Week 52)	Clinical remission per mMS (Week 40)
**CD induction**	CDAI remission + endoscopic response (Week 12); CDAI remission + endoscopic response (Week 52, Study 1)	CDAI remission + endoscopic response (Week 52, SIBERITE-1); CDAI remission + endoscopic response (Week 12, SIBERITE-2)	CDAI remission (CDAI and PRO-2) + endoscopic response (Week 12)
**CD maintenance**	Integrated (Week 52 endpoints in induction protocol)	Integrated (Week 52 endpoints in SIBERITE-1)	CDAI remission (CDAI and PRO-2) + endoscopic response (Week 40)
**Co-primary structure**	Parallel U.S./FDA and EU/EMA endpoints in CD	Biomarker-defined subgroup analyses as secondary endpoints	Parallel U.S./FDA (CDAI) and EU/EMA (PRO-2) co-primary endpoints in CD
**Sample size, timeline and global representation**			
***N* (UC program), estimated**	1020	750 (AMETRINE-1: 400; AMETRINE-2: 350)	1651 (SUNSCAPE-1: 980; SUNSCAPE-2: 671)
***N* (CD program), estimated**	1200	1025 (SIBERITE-1: 600; SIBERITE-2: 425)	1651 (STARSCAPE-1: 980; STARSCAPE-2: 671)
**Recruitment start**	UC: October 2023 CD: June 2024	UC: September 2024 CD: March 2025	UC: October 2025 CD: October 2025
**Est. study completion**	UC: August 2029 CD: November 2029	UC: December 2029 CD: December 2033	UC: April 2033 CD: March 2034
**Sites**	>950	>630	≈450
**Countries**	>40	≈35	>30
**Est. completion**	UC: August 2029; CD: November 2029	2027-2033	2028-2034

All data sourced exclusively from publicly registered ClinicalTrials.gov record; accessed February 2026. No protocol-derived or confidential information is included.

Abbreviations: CDAI, Crohn’s Disease Activity Index; CD, Crohn’s disease; IV, intravenous; mMS, modified Mayo Score; MSD, Merck Sharp & Dohme; OLE, open-label extension; PRO-2, 2-item patient-reported outcome; RCT, randomized controlled trial; SC, subcutaneous; UC, ulcerative colitis.

### Tulisokibart (MK-7240)

Tulisokibart, originally developed by Prometheus Biosciences (acquired by MSD for U.S.$10.8 billion in 2023) under the designation PRA023, is a humanized IgG1κ monoclonal antibody that binds both soluble and membrane-bound TL1A.^54^ The molecule is now in phase 3 development for both UC and CD.

#### ARTEMIS-UC (phase 2, ulcerative colitis)

The phase 2a ARTEMIS-UC trial randomized 135 adults with moderately to severely active UC (modified Mayo score [MMS] 5-9, endoscopic subscore ≥ 2) to intravenous tulisokibart (1000 mg day 1, 500 mg weeks 2, 6, 10) or placebo.^13^ Patients were glucocorticoid-dependent or had inadequate response to conventional. Up to 47% of patients were previously exposed to advanced therapies. Enrollment employed a precision medicine strategy incorporating a genetic diagnostic test; cohort 1 enrolled regardless of biomarker status, while cohort 2 enrolled only biomarker-positive patients.

At week 12, the primary endpoint of clinical remission (rectal bleeding subscore 0, stool frequency subscore ≤ 1) was achieved by 26% of tulisokibart-treated patients vs 1% placebo in cohort 1 (difference 25 percentage points; 95% CI 14-37; *P* < .001). Endoscopic improvement (endoscopic subscore ≤ 1 without friability) occurred in 37% in the tulisokibart group vs 6% in the placebo group (difference 30 percentage points; *P* < .001). All ranked secondary endpoints significantly favored tulisokibart, including clinical response, histological improvement, combined endoscopic-histological improvement, and symptomatic remission. Extended analysis of symptomatic endpoints demonstrated that tulisokibart-treated patients achieved higher rates of stool frequency normalization (57.4% vs 17.9%), rectal bleeding resolution (61.8% vs 34.3%), and symptomatic remission (47.1% vs 13.4%) compared with placebo at week 12, with improvements observed as early as week 2. Among week 12 responders receiving 250 mg maintenance, symptomatic remission (68.0%) and disease clearance, defined as symptomatic remission plus mucosal healing, (44.0%) continued to increase through week 50.^55^ Biomarker-stratified results from this trial are discussed in the Genetic Associations and Precision Medicine section below. The open-label extension demonstrated maintenance of efficacy through week 50 in induction responders, with the 250 mg subcutaneous maintenance dose showing numerically greater durability than 100 mg.^56^

#### APOLLO-CD (phase 2a, Crohn’s disease)

The APOLLO-CD trial was an open-label, proof-of-concept, single-arm study enrolling 55 adults with moderate-to-severe CD [Crohn’s Disease Activity Index (CDAI) 220-450, Simple Endoscopic Score for Crohn’s Disease (SES-CD) ≥ 6 or ≥ 4 for isolated ileal disease].^17^ Patients had high rates of prior biologic exposure (71%) and an average disease duration of 10.3 years, representing a refractory population. Tulisokibart was administered intravenously at 1000 mg on day 1, followed by 500 mg at weeks 2, 6, and 10.

The primary endpoint of endoscopic response (≥50% reduction in SES-CD) at week 12 was achieved by 26% of patients vs 12% historical placebo estimate (difference 14 percentage points; *P* = .002). Clinical remission (CDAI < 150) was observed in 49% vs a 16% historical placebo estimate (*P* < .001). Among biologic-experienced patients, 33% achieved endoscopic response and 39% achieved clinical remission, indicating preserved efficacy in this difficult-to-treat population. Significant reductions in C-reactive protein and fecal calprotectin supported objective evidence of anti-inflammatory activity.

#### Phase 3 program

MSD (Merck Sharp & Dohme) has initiated comprehensive phase 3 programs: ATLAS-UC (NCT06052059) in UC and ARES-CD (NCT06430801) in CD.^57^ These represent the first phase 3 clinical studies for any anti-TL1A antibody in IBD. Primary completion is anticipated in 2026, with final results expected to inform regulatory submissions. Additionally, phase 2 development is ongoing in systemic sclerosis-associated ILD (ATHENA-SSc-ILD, NCT05270668), and phase 2b trials have been initiated in hidradenitis suppurativa (NCT06956235), radiographic axial spondyloarthritis (NCT07133633), and rheumatoid arthritis (NCT07176390).^58^

### Afimkibart (RO7790121)

Afimkibart, formerly PF-06480605, was developed by Pfizer, subsequently partnered with Roivant Sciences, and is now being advanced by Roche/Genentech following acquisition. The molecule is a fully human IgG1 monoclonal antibody that binds both soluble and membrane-bound TL1A, preventing interaction with DR3.^59^

#### Phase 1 and TUSCANY phase 2a

A first-in-human phase 1 study established favorable pharmacokinetics, pharmacodynamics, and tolerability across single and multiple ascending intravenous doses (up to 800 mg and 500 mg, respectively) in healthy subjects.^60^ The TUSCANY phase 2a trial was an open-label trial treated 50 patients with moderate-to-severe UC with intravenous afimkibart 500 mg every two weeks for seven doses; 38% achieved endoscopic improvement (MES 0 or 1) at week 14. Colonic transcriptomic analysis confirmed reduced expression of inflammatory and fibrosis-related genes.^51^ These findings were confirmed in a placebo-controlled TUSCANY-2 serum proteomic analysis using a 250-protein NULISAseq panel: afimkibart significantly downregulated 45 proteins spanning Th17 (IL-17A/F), myeloid (G-CSF and IL-6), and fibrosis-related (MMP8, MMP12, and HGF) pathways. Oncostatin M (OSM), a marker of anti-TNF nonresponse, was also suppressed, suggesting mechanistic differentiation from TNF inhibition.^61^

#### TUSCANY-2 phase 2b

TUSCANY-2 was a multicenter, double-blind, placebo-controlled phase 2b study across 114 centers in 23 countries.^14^ In total, 246 adults with moderately to severely active UC (total Mayo score 6-12, endoscopic subscore ≥ 2) were randomized to subcutaneous afimkibart 50 mg, 150 mg, or 450 mg every four weeks for a 12-week induction followed by maintenance.

The primary endpoint of clinical remission by total Mayo score (tMS ≤ 2, no individual subscore > 1) at week 14 was not statistically significant for any dose vs placebo (26%, 23%, 24% vs 12% for 50 mg, 150 mg, 450 mg, and placebo, respectively). However, by the modified Mayo score (current FDA-preferred endpoint), clinical remission rates were 30% (50 mg), 35% (150 mg), and 32% (450 mg) vs 12% (placebo), with endoscopic improvement and fecal calprotectin normalization also favoring afimkibart. The primary endpoint miss has been attributed to a higher-than-anticipated placebo response (12% vs the assumed 6%) and the inclusion of the subjective Physician Global Assessment in the total Mayo score.

In the treat-through maintenance period, efficacy was sustained through week 56 across all dose groups.^14^ Clinical remission by mMS was achieved by 37.9% (50 mg), 38.5% (150 mg), and 35.7% (450 mg) at week 14. Endoscopic improvement at week 56 was 38.1% (50 mg), 39.3% (150 mg), and 35.7% (450 mg). Histologic-endoscopic mucosal improvement at week 14 was 30% (50 mg), 32% (150 mg), and 26% (450 mg) vs 5% placebo, sustained at week 56. The safety profile remained acceptable, with 132 (59%) of 224 patients reporting at least one treatment-emergent adverse event and no deaths.

Biomarker-stratified analyses are discussed in a later section. Based on these data, phase 3 programs have been initiated, including AMETRINE-1 (NCT06589986) and AMETRINE-2 (NCT06588855) for ulcerative colitis, and SIBERITE-1 (NCT06819878) and SIBERITE-2 (NCT06819891) for Crohn’s disease.

### Duvakitug (TEV-48574)

Duvakitug (Teva/Sanofi) is a human IgG1 monoclonal antibody blocking TL1A–DR3 binding.^15^ It is the only anti-TL1A agent evaluated in a concurrent UC and CD basket design, employing an all-comer population stratified by prior advanced therapy exposure.

#### Phase 1

Phase 1 studies in healthy volunteers and mild-asthma patients established dose-proportional pharmacokinetics with minimal accumulation on biweekly dosing.^62^ Free TL1A suppression persisted up to two months after the last dose even when drug levels were low or undetectable, suggesting prolonged pharmacodynamic effects. Safety was favorable with good tolerance up to 2300 mg.

#### RELIEVE UCCD phase 2b

RELIEVE UCCD was a 14-week phase 2b randomized, double-blind, dose-ranging basket study in adults with moderately to severely active UC or CD with inadequate response, loss of response, or intolerance to conventional or advanced therapies.^15^^,^^16^ The study incorporated a Bayesian design. Patients received a subcutaneous duvakitug 2250 mg loading followed by 450 mg or 900 mg every two weeks vs placebo.

In UC (*n* = 137), both doses met the primary endpoint of clinical remission (MMS ≤ 2) at week 14: 36% (450 mg) and 48% (900 mg) vs 20% placebo.^15^ These are among the highest remission rates reported for any novel-mechanism IBD therapy. Efficacy was observed regardless of prior advanced therapy exposure.

In CD (*n* = 138), the primary endpoint of endoscopic response (≥50% reduction in SES-CD) at week 14 was achieved by 26% (450 mg) and 48% (900 mg) vs 13% placebo, with posterior mean differences versus placebo of 13% (95% CrI -3 to 29) and 33% (95% CrI 16 to 50), respectively.^16^ Both doses demonstrated superiority to placebo on prespecified Bayesian analysis (>0.90 posterior probability), with a treatment effect observed in both advanced therapy-experienced and therapy-naive patients. Adverse events occurred in 49% (450 mg), 43% (900 mg) and 52% (placebo) of the UC cohort, and in 67%, 43% and 48% of the CD cohort; adverse events leading to discontinuation were uncommon throughout (UC 0%, 2% and 5%; CD 9%, 2% and 2%, respectively). An open-label extension (NCT05668013) is evaluating maintenance efficacy.

Long-term extension data presented in February 2026 demonstrated durable clinical and endoscopic efficacy maintained over 44 weeks in induction responders in both UC and CD, supporting the ongoing phase 3 STARSCAPE and SUNSCAPE programs.^63^

### Differential effects on DcR3

Anti-TL1A antibodies differ in their effect on the interaction between TL1A and its decoy receptor DcR3. Afimkibart inhibits TL1A binding to DcR3, whereas duvakitug binds a TL1A epitope centered on residue R32 that is critical for engagement of DR3 but not DcR3, thereby preferentially inhibiting TL1A–DR3 signaling while largely preserving TL1A–DcR3 binding;^64^ the effect of tulisokibart on DcR3 has not been explicitly reported. Because DcR3 also neutralizes Fas ligand and LIGHT, preservation of DcR3 function has been proposed as potentially advantageous.^65^ However, an exploratory analysis from the TUSCANY program presented as a conference abstract found that DcR3 concentrations did not correlate with response to afimkibart and that afimkibart did not diminish DcR3-mediated neutralization of Fas ligand and LIGHT, suggesting that any disruption of TL1A–DcR3 binding may be biologically inconsequential; these data are preliminary pending full publication.^66^

### Integrated safety assessment

Across clinical development programs, TL1A inhibitors have demonstrated a reassuringly favorable safety profile.^13–17^^,^^67^ No serious treatment-emergent adverse events attributable to TL1A inhibition have been consistently identified, and the theoretical concern that blocking a TNF superfamily member might increase infection risk has not been borne out, with serious infection rates comparable between active and placebo arms.

Immunogenicity profiles have varied across TL1A inhibitor programs. The early PF-06480605 formulation (precursor to afimkibart) showed high ADA rates (82%), potentially related to bivalent antibody–trimeric TL1A immune complex formation, though without significant efficacy impact at therapeutic doses.^60^ Current optimized molecules show more favorable immunogenicity, with inverse dose-response relationships for afimkibart (minimal ADA impact at higher doses) and duvakitug (ADA mainly at lower doses with tolerance at higher doses). These findings contrast with TL1A-directed bispecifics, where AMG966 (Amgen) and RG6730 (Roche) showed 100% ADA formation with > 80% neutralizing antibodies, leading to program discontinuation.^68^ The monoclonal antibody format thus appears well-suited to TL1A targeting with appropriate dose optimization, though long-term phase 3 and post-marketing surveillance will be essential to characterize rare adverse events.

### Global recruitment and population diversity

The phase 3 development programs for TL1A inhibitors present a unique opportunity to redress longstanding disparities in IBD clinical trial representation.^69^ Unlike earlier generations of biologics, whose pivotal datasets derived predominantly from North American and Western European centers, the current TL1A inhibitor trials have established recruitment networks extending substantially into Latin America, East Asia, and the Middle East.^70^ The tulisokibart program encompasses over 950 sites across more than 40 countries spanning six continents;^71^^,^^72^ the afimkibart program involves over 630 sites in approximately 30 countries, including India and South Africa;^73–76^ and the duvakitug program follows a comparable global footprint.^77–80^ This geographic expansion reflects both recruitment necessity and independent scientific value by enhancing the generalizability of trial findings, a consideration particularly germane to TL1A inhibitors where companion diagnostic strategies rely upon TNFSF15 polymorphisms whose prevalence differs across ancestral populations.^81^ Validating efficacy signals and biomarker associations across diverse cohorts would establish TL1A inhibitors as a model for global precision medicine in IBD.

## Genetic associations and precision medicine

Having reviewed the clinical evidence for TL1A inhibition, we now turn to the genetic underpinnings that may enable precision targeting of this pathway.

### TNFSF15 as an IBD risk and severity locus

Genome-wide association studies (GWAS) have consistently identified TNFSF15 as a susceptibility locus for IBD across diverse ancestral populations.^82–84^ Initially discovered in Japanese CD cohorts, the association has been replicated in European, African American, and other groups, with effect sizes among the largest for common IBD risk variants. A meta-analysis of 15 studies (8903 CD, 4687 UC, 12606 controls) identified six SNPs associated with CD and three with UC, with certain polymorphisms showing ancestry-specific associations.^85^ Three haplotypes have been defined: A (high-risk), B (protective across Japanese, European, and U.S. populations), and C (neutral).^82^^,^^86^ A transethnic study of 86 640 European and 9846 East-Asian, Indian, or Iranian patients showed that TNFSF15 heterogeneity is driven by effect size rather than allele frequency, raising the possibility of East Asian-specific gene–environment interactions.^87^ Genetic associations are more ancestry-dependent in CD than UC.^88^ Recent transancestry meta-analysis confirms TNFSF15 associations and identifies a novel TNFRSF25 (DR3) SNP as an IBD-specific polymorphism, suggesting genetic variation in both ligand and receptor may influence susceptibility.^84^

Importantly, TNFSF15 polymorphisms associate not only with susceptibility but also with disease severity and phenotype. Patients harboring risk haplotypes show a more complicated course, including higher rates of fibrostenotic complications and stenosis-associated surgeries in CD.^85^^,^^89^ These observations suggest that patients with genetically elevated TL1A pathway activity may derive particular benefit from TL1A-targeted therapy, forming the conceptual basis for companion diagnostic development.

### Genetic biomarker development

A distinguishing feature of the TL1A inhibitor development program has been the parallel advancement of genetic-based companion diagnostics (CDx). The hypothesis that individuals harboring genetic signatures associated with heightened TL1A activity might demonstrate superior response to TL1A inhibition has been incorporated into clinical trial design from the outset.^90^

In the phase 2 ARTEMIS-UC trial of tulisokibart, a prespecified genetic biomarker algorithm was employed to stratify patients by predicted likelihood of response.^13^ In the combined biomarker-positive population from both cohorts, clinical remission rates were 32% vs 11% for tulisokibart vs placebo (difference 21 percentage points; 95% CI, 2-38; *P* = .02), compared with 26% vs 1% in the mixed population of cohort 1 (difference 25 percentage points), warranting cautious interpretation given the modest numerical magnitude. In the TUSCANY-2 trial of afimkibart, biomarker-positive patients demonstrated numerically greater treatment benefit for endoscopic improvement (41.6% vs 24.0% risk difference vs placebo), though the trial was not powered for definitive biomarker assessment.^14^ Similar biomarker stratification approaches have been employed in the duvakitug program.^15^

Several challenges remain in validating genetic companion diagnostics across diverse populations. The incremental benefit of genetic enrichment in ARTEMIS-UC was numerically lower in biomarker-positive patients (21 percentage point treatment difference) than in the mixed population in cohort 1 (25 percentage points), though this may reflect a higher placebo response in the biomarker-positive group (11% vs 1%) rather than a diminished drug effect.^13^ The cohort 2 validation population, enrolling exclusively biomarker-positive patients, showed weaker discriminatory capacity than cohort 1, raising questions about the clinical utility of the companion diagnostic. GWAS associations for TNFSF15 are more robust in CD than UC, with genetic associations more ancestry-dependent in CD.^85^^,^^88^ Companion diagnostic performance may therefore differ between diseases, requiring validation in appropriately powered phase 3 studies across ancestral groups.

In contrast, duvakitug achieved its highest efficacy rates in RELIEVE UCCD without companion diagnostic enrichment, using an unselected all-comer population stratified only by prior advanced therapy exposure.^15^^,^^16^ This raises the possibility that TL1A inhibition may be effective regardless of TNFSF15 genotype, with implications for clinical applicability if regulatory approval does not mandate a companion diagnostic. While the benefit of genetic selection remains to be established in phase 3 trials, this precision medicine approach is notable in IBD drug development. If validated, TL1A inhibitors may become the first IBD class with approved companion diagnostics, enabling genotype-guided selection and potentially improving cost-effectiveness.^91^^,^^92^ However, given that the initial ARTEMIS-UC companion-diagnostic results showed only a numerically small incremental benefit from genetic enrichment, phase 3 readouts will be the definitive test of whether genotype-guided selection is clinically meaningful for this class.

### Therapeutic timing and speed of onset

Whether anti-TL1A therapy should be positioned early or reserved for biologic-experienced patients remains unresolved. A pooled subgroup meta-analysis of ARTEMIS-UC, TUSCANY-2, and RELIEVE-UCCD found that advanced-therapy-experienced patients derived numerically larger relative benefit (pooled relative risk 5.04 vs 1.84 in RELIEVE cohorts), although the interaction did not reach statistical significance.^93^ First-line positioning will require head-to-head trials, validated biomarkers, durable safety data, and cost-effectiveness evidence. The antifibrotic mechanism could favor earlier use; fibrosis is established early in stricturing CD and is essentially irreversible, but this hypothesis remains untested. Speed of onset is underexplored. Because TL1A signals upstream of TNF, JAK-STAT, and IL-13 pathways, clinical response could be rapid; exploratory ARTEMIS-UC data showed early separation from placebo by week 4. No anti-TL1A program has enrolled acute severe UC or performed intravenous induction dose-finding in this population, but a proof-of-concept study would test whether upstream blockade translates to faster clinical benefit.

## Emerging pipeline and next-generation approaches

The TL1A inhibitor landscape has expanded dramatically beyond the three established agents, with at least 16 additional programs in clinical development and substantial innovation in antibody engineering, bispecific formats, and alternative targeting strategies (Table S1).^18^^,^^94^

### Extended half-life antibodies

First-generation TL1A inhibitors require dosing every 2-4 weeks. Next-generation molecules are engineered for extended half-lives, potentially enabling quarterly or twice-yearly dosing with implications for patient convenience and adherence.

#### SPY002 (Spyre therapeutics)

Phase 1 interim results reported in June 2025 demonstrated a ∼75-day half-life, more than three times longer than first-generation competitors.^95^ At doses as low as 100 mg, complete TL1A suppression was achieved through 20 weeks with no serious adverse events. Spyre has initiated the phase 2 SKYLINE-UC trial testing SPY002 as monotherapy and in combinations with SPY001 (anti-α4β7) and SPY003 (anti-IL-23).^96^

#### XmAb942 (Xencor)

Leveraging proprietary Xtend™ Fc technology, XmAb942 is an extended half-life anti-TL1A antibody with high-class potency. Phase 1 SAD/MAD data (presented as a conference abstract) confirmed a terminal half-life >74 days, dose-proportional pharmacokinetics across IV and subcutaneous routes, and suppression of free soluble TL1A below the limit of quantification for 140-196 days, supporting infrequent subcutaneous maintenance dosing.^97^^,^^98^ The phase 2b XENITH-UC trial in moderately to severely active UC is now enrolling.

#### ABS-101 (Absci)

Represents the first AI-designed anti-TL1A antibody to enter clinical trials, developed using Absci’s de novo generative AI foundation model.^99^ The molecule progressed from program initiation to IND filing in approximately two years, substantially faster than industry standards. Phase 1 dosing began in May 2025.

#### FG-M701 (AbbVie/FutureGen)

Licensed by AbbVie in June 2024 for U.S.$150 million upfront plus up to U.S.$1.56 billion in milestones.^100^ The fully human monoclonal antibody is engineered for extended dosing intervals, signaling AbbVie’s strategic commitment to the TL1A space.

#### BCD-261 (Biocad)

A next-generation anti-TL1A monoclonal antibody developed by Biocad (Russia), currently being evaluated in two phase 2 trials: BCD-261-2/COMANDOR in moderate-to-severe Crohn’s disease (planned enrollment 510 patients) and BCD-261-4/ULTRAMARINE (NCT07080034) in moderate-to-severe ulcerative colitis (planned enrollment 495 patients), each enrolling adults with inadequate response to glucocorticoids, immunosuppressants, or prior biologics.^101^^,^^102^ BCD-261 expands the geographic and competitive landscape of TL1A blockade beyond the major Western and East Asian programs and is particularly relevant for Eurasian healthcare systems.

### Bispecific antibodies

Bispecific antibodies targeting TL1A alongside established IBD pathways represent the most innovative approach to overcoming the therapeutic ceiling, potentially achieving additive or synergistic efficacy through simultaneous pathway inhibition.

#### CLD-423 (Caldera therapeutics)

The first-in-class bispecific targeting both IL-23p19 and TL1A entered phase 1 in early 2026.^103^ The rationale combines established IL-23 inhibition (demonstrated by risankizumab and guselkumab) with TL1A’s unique antifibrotic properties. Caldera launched with U.S.$112.5 million funding from leading healthcare investors.

#### Xencor TL1A × IL-23p19 (XmAb412)

A native-like bispecific incorporating Xtend™ half-life extension, with single-digit picomolar TL1A affinity and sub-picomolar IL-23 affinity. Allometric scaling predicts a human half-life of ≥60 days. First-in-human studies are planned for 2026.^104^

#### RO7837195 (Roche; formerly PF-07261271, Pfizer)

Targets p40 (shared subunit of IL-12 and IL-23) combined with TL1A, potentially affecting both IL-12 and IL-23 pathways, unlike p19-selective approaches.^105^ Phase 1 results in 35 healthy volunteers demonstrated a favorable safety profile, with dose-dependent increases in total soluble TL1A and p40 confirming dual target engagement and no apparent impact of antidrug antibodies on pharmacokinetics or pharmacodynamics.^106^ RO7837195 is being evaluated in a phase 2b UC trial (NCT06979336).

#### SIM0709 (Simcere/Boehringer Ingelheim)

In January 2026, Boehringer Ingelheim licensed SIM0709, a long-acting TL1A/IL-23p19 bispecific antibody, from Simcere Pharmaceuticals for up to €1.05 billion (U.S.$1.26 billion) in total potential payments.^107^ In preclinical studies, SIM0709 demonstrated superior synergistic efficacy exceeding even the combination of the corresponding monotherapies in both in vitro primary cell assays and in vivo animal models.^108^ This transaction represents the second billion-dollar bispecific licensing deal in the TL1A space within a year, following the Earendil/Sanofi agreement.

#### Chinese biotechnology innovations

Multiple bispecific formats are emerging, including HXN-1003 (TL1A/IL-23 p19 bispecific), HXN-1002 (alpha4beta7/TL1A bispecific with unique mechanism enhancing integrin internalization), and LQ080 (TL1A/IL-23 p19 single-domain biparatopic antibody with extended half-life).^109–111^ HXN-1002 and HXN-1003, developed by Earendil Labs/Helixon Therapeutics, were licensed to Sanofi in April 2025 for U.S.$125 million upfront plus up to U.S.$1.72 billion in development and commercial milestones, representing significant corporate validation of the TL1A bispecific strategy.^112^ Additionally, ALX001, a TL1A/IL-23 bispecific antibody with a symmetric 2 + 2 format, has demonstrated preclinical superiority over constituent monotherapies in mouse colitis and psoriasis models, with a favorable pharmacokinetic profile in non-human primates.^113^

#### Limitations with bispecific antibodies

The development of TL1A-directed bispecific antibodies has been complicated by significant immunogenicity challenges. Previous programs including, AMG966 (Amgen) and RG6730 (Roche), demonstrated 100% antidrug antibody formation with greater than 80% neutralizing antibodies, leading to program discontinuation. This immunogenicity burden may explain why current bispecific development is proceeding cautiously, with CLD-423 employing novel engineering approaches to mitigate this risk. The immunogenicity challenge also provides theoretical rationale for alternative approaches such as DR3 receptor antagonism (SL-325), which avoids targeting the soluble ligand and the potential for immune complex formation that may drive antidrug antibody responses.

### Oral TL1A modalities

Preclinical data have been reported for a first-in-class oral anti-TL1A nanobody that selectively blocks TL1A–DR3 while sparing TL1A–DcR3, alongside a long-acting subcutaneous TL1A/IL-23 bispecific nanobody. The oral molecule was stable in simulated gastrointestinal fluid, distributed preferentially to intestinal tissue with minimal systemic exposure, and was active in murine DSS colitis.^114^ A gut-restricted oral anti-TL1A agent could uncouple efficacy from systemic immunosuppression, with relevance for younger patients, infection-prone individuals, and long-term maintenance. Oral small molecule approaches face a distinct challenge: unlike cytokine receptors with defined enzymatic active sites, the TL1A–DR3 interface is a broad protein–protein interaction surface inherently difficult to target with conventional chemistry. Nimble Therapeutics and PsiThera are pursuing early-stage oral small molecule programs, though clinical data remain limited.^114^

### DR3 receptor antagonism

#### SL-325 (Shattuck labs)

Represents the first clinical-stage drug targeting DR3 rather than TL1A, offering an alternative mechanistic approach.^115^ The FDA cleared the IND in August 2025, with phase 1 dosing initiated in Q3 2025. DR3 targeting offers theoretical advantages including potentially reduced immunogenicity (TL1A antibodies form immune complexes that may drive antidrug antibody formation), while DR3 remains membrane-bound. SL-325 binds DR3 with picomolar affinity (1.36 pM) and blocks TL1A with an IC50 of 0.06 nM. Shattuck secured U.S.$103 million in financing to fund development into 2029.

## Discussion and future perspectives

### Positioning TL1A inhibitors in the IBD treatment landscape

TL1A inhibitors arrive at a time of therapeutic abundance in IBD, with gastroenterologists facing unprecedented choices among TNF inhibitors, integrin antagonists, IL-12/23, IL-23 blockers, S1PRM, and JAK inhibitors. The central question confronting physicians and patients is whether TL1A inhibition offers advantages that justify its selection over established mechanisms.

Based on available phase 2 data, efficacy appears similar to existing biologics, with clinical remission rates comparable to those observed for IL-23 inhibitors and integrin antagonists. While cross-trial comparisons require cautious interpretation, placebo-adjusted treatment differences show consistency across programs, ranging from approximately 18 to 33 percentage points depending on the agent, dose, and endpoint.^13–16^ These comparisons await resolution through adequately powered phase 3 trials. Notably, TL1A inhibitors have demonstrated preserved efficacy in biologic-experienced populations, a critical attribute given that sequential biologic failure is increasingly common in clinical practice.

Differences in phase 3 trial design will influence interpretation of future results. Afimkibart’s treat-through design, in which all randomized patients continue on the same regimen from induction through maintenance, differs fundamentally from tulisokibart’s integrated induction-maintenance approach within a single protocol and from duvakitug’s separate induction and maintenance study design incorporating responder re-randomization, in which only clinical responders enter the maintenance trial. Duvakitug’s transition from a Bayesian adaptive phase 2 framework to a frequentist phase 3 design is also notable. These methodological differences, alongside variations in patient populations, endpoint definitions, and co-primary structures (with both tulisokibart and duvakitug employing parallel U.S./FDA and EU/EMA endpoints in Crohn’s disease), should be carefully considered when comparing outcomes across programs.

The dual anti-inflammatory and potentially antifibrotic mechanism represents the most compelling differentiator. If confirmed in phase 3 trials and real-world studies, the capacity to address fibrotic disease progression could position TL1A inhibitors as preferred therapy for patients with inflammatory strictures CD and potentially for those with long-standing UC at risk of submucosal fibrosis. The ongoing ATHENA-SSc-ILD trial will provide important data on antifibrotic activity in a disease setting where fibrosis is the primary pathological process.

### Combination therapy and treatment sequences

The emergence of bispecific antibodies and the development of extended half-life molecules enabling combination strategies herald a potential shift toward multi-pathway targeting in IBD. The VEGA trial demonstrating enhanced efficacy with guselkumab plus golimumab, along with the ongoing phase 2b trials DUET-CD (NCT05242471) and DUET-UC (NCT05242484) evaluating this combination at multiple dose levels, provides proof-of-concept for IL-23 plus TNF combination therapy, with obvious implications for TL1A-containing combinations.^116^

Preclinical data now provide direct evidence supporting TL1A-based combination approaches. In the anti-CD40 mouse colitis model, the combination of anti-TL1A and anti-IL-23 antibodies was significantly more efficacious than either monotherapy across body weight loss, disease activity scores, colon weight-to-length ratio, and histopathology.^117^

These preclinical findings are being translated clinically through SKYLINE-UC, the first platform study in UC to evaluate three long-acting antibodies (anti-alpha4beta7 [SPY001], anti-TL1A [SPY002], and anti-IL-23 [SPY003]) as monotherapies and pairwise combinations within a single master protocol.^96^ The platform design claims to reduce sample size by an estimated 45% compared with separate studies, with Part A data anticipated in 2026 and Part B in 2027. Spyre’s broader strategy of developing these extended half-life antibodies with the explicit goal of high-concentration coformulation supporting single-injection multi-pathway therapy represents an ambitious vision for next-generation IBD treatment. Whether such approaches will prove necessary or cost-effective remains to be determined, but they reflect recognition that monotherapy may be insufficient for a substantial proportion of patients.

### Resistance, patient selection, and combination strategies

Although anti-TL1A agents have shown consistent phase 2 efficacy, several practical questions will determine their optimal use. Primary and secondary resistance mechanisms remain speculative but are likely to resemble those of other advanced therapies, including escape through redundant effector pathways such as TNF, IL-23, and JAK-STAT signaling, and secondary loss of response mediated by antidrug antibodies. Optimal patient selection may ultimately depend on biomarkers beyond TNFSF15 genotype, such as circulating and mucosal TL1A concentrations, serum proteomic signatures, and tissue transcriptomic profiles, although none is yet validated for treatment selection. Finally, the anticipated ceiling of monotherapy efficacy provides a rationale for combination strategies with established advanced therapies; the bispecific constructs and platform trials discussed above, together with the proof of concept provided by the VEGA study,^116^ illustrate how TL1A blockade might be combined with IL-23 or other mechanisms to extend the therapeutic ceiling.

### TL1A blockade beyond IBD: an unusual lead indication

Unusually for a novel immunology mechanism, IBD is the most advanced clinical indication for anti-TL1A blockade. The furthest non-IBD programs remain in phase 2: tulisokibart in systemic sclerosis-associated interstitial lung disease, hidradenitis suppurativa, axial spondyloarthritis, and rheumatoid arthritis; afimkibart in moderate-to-severe asthma; and duvakitug in asthma and eosinophilic oesophagitis.^58^ This sequence reflects the strong IBD-specific genetic signal at TNFSF15 and the tissue-agnostic role of TL1A in inflammation and fibrosis. Success beyond IBD would broaden the commercial opportunity and potentially enable therapeutic selection based on extraintestinal manifestations, though whether the class will demonstrate equivalent benefit in non-IBD indications remains an open question.

### Limitations and uncertainties

Several important uncertainties await resolution. First, phase 3 efficacy must be confirmed, as the history of IBD drug development includes numerous programs that failed to replicate promising phase 2 results. Second, the degree of antifibrotic benefit achievable in established fibrostenotic disease remains speculative; prevention of fibrosis progression may be more achievable than reversal of established strictures, but it is extremely challenging to study within conventional drug development. Third, long-term safety, particularly regarding infection risk and malignancy given TL1A’s roles in host defense and tumor surveillance, requires extended observation. From a practical standpoint, the optimal positioning of TL1A inhibitors relative to IL-23 blockers (which share some mechanistic overlap through suppression of Th17 responses) and integrin antagonists will require head-to-head comparative effectiveness studies and real-world evidence generation.

Several high-unmet-need IBD populations remain unexplored in TL1A inhibitor development despite strong mechanistic rationale. No dedicated pediatric trials have been initiated, though Roche has announced planned studies in pediatric UC (NCT07158242) and CD (SIBERITE-PEDS). Fistulizing Crohn’s disease, affecting a significant proportion of patients with achievement of long-term healing in only 30%-50% with current anti-TNF therapy, has not been specifically targeted despite TL1A’s antifibrotic mechanism being theoretically appealing for promoting fistula healing. Pouchitis, developing in patients following IPAA surgery for UC, and post-surgical recurrence prevention represent additional opportunities where *TNFSF15* haplotypes associated with higher TL1A production are linked to increased stricturing behavior and surgical need.^89^

## Conclusions

TL1A inhibitors represent the culmination of over two decades of translational research, evolving from target identification in 2002 through genetic validation, mechanistic characterization, and now late-stage clinical development. Phase 2 trials have established proof-of-concept for TL1A blockade in both UC and CD, with efficacy observed in biologic-experienced populations and favorable safety profiles.

The dual anti-inflammatory and antifibrotic mechanism, the potential for precision medicine through companion diagnostics, and the robust pipeline of next-generation molecules collectively position TL1A inhibitors as potentially transformative for IBD therapeutics. Phase 3 results anticipated in 2026-2027 will determine whether these agents fulfill their promise of addressing the unmet needs that persist despite the current biological armamentarium. A recent MSD press release has reported provisional top-line results that tulisokibart met its primary and key secondary endpoints in the phase 3 ATLAS-UC induction study, with safety consistent with phase 2; no efficacy data have yet been released, and this unpublished announcement should be interpreted with considerable caution pending full peer-reviewed results.^118^

For patients with an inflammatory stricturing CD phenotype, chronic UC with fibrotic complications, or those refractory to existing mechanisms, TL1A inhibitors may offer a promising novel therapeutic avenue. For the field of IBD drug development, this class demonstrates that disciplined translational science from genetics through biology to clinical development can yield therapeutic innovation even in an era of perceived target exhaustion.

## Supplementary Material

izag172_Supplementary_Data

## Data Availability

No original data were generated or analyzed in the preparation of this article. This is a narrative review; all data discussed are derived from published literature, publicly available clinical trial registry records, and conference abstracts, as cited in the reference list.
