# Supplementary material for: Targeting the TL1A pathway in inflammatory bowel disease: mechanistic insights and emerging therapeutic pipeline
Source: Inflamm Bowel Dis. 2026 Aug 27;32(11):2212–31. doi: 10.1093/ibd/izag172 (PMC13722117; doi:10.1093/ibd/izag172)
Supplement: Supplementary file 1 [file izag172_supplementary_data.docx]

**Supplementary Materials**

*Targeting the TL1A Pathway in Inflammatory Bowel Disease: Mechanistic Insights and Emerging Therapeutic Pipeline*

*Mohammed Nabil Quraishi, Vipul Jairath, Badr Al-Bawardy*

**Contents**

Figure S1. PRISMA 2020 flow diagram for the literature search.

Table S1. TL1A-targeted therapeutics in clinical and preclinical development for inflammatory bowel disease.

**Figure S1. PRISMA 2020 flow diagram for the literature search.**

An electronic search was conducted in MEDLINE, Embase, and the Cochrane Central Register of Controlled Trials from inception to 16 May 2026 using the following search strategy: (TL1A[TIAB] OR TNFSF15[TIAB] OR DR3[TIAB] OR TNFRSF25[TIAB]) AND (inflammatory bowel disease[TIAB] OR ulcerative colitis[TIAB] OR Crohn's disease[TIAB]) AND (tulisokibart[TIAB] OR afimkibart[TIAB] OR duvakitug[TIAB] OR TEV-48574[TIAB] OR MK-7240[TIAB] OR PRA023[TIAB] OR PF-06480605[TIAB] OR RVT-3101[TIAB] OR RO7790121[TIAB] OR XmAb942[TIAB] OR XmAb412[TIAB] OR SPY002[TIAB] OR BCD-261[TIAB] OR PF-07261271[TIAB] OR RO7837195[TIAB] OR CLD-423[TIAB] OR SIM0709[TIAB] OR HXN-1002[TIAB] OR HXN-1003[TIAB] OR LQ080[TIAB] OR ALX001[TIAB] OR FG-M701[TIAB] OR ABS-101[TIAB] OR SL-325[TIAB] OR anti-TL1A[TIAB] OR TL1A inhibit*[TIAB] OR DR3 antagonist[TIAB]) NOT (review[PT] OR letter[PT]). This primary search yielded 985 articles. Additionally, the reference lists of selected articles, systematic reviews, and meta-analyses were reviewed to identify further studies of potential relevance, including recent conference abstracts from major gastroenterology meetings (Digestive Disease Week, European Crohn's and Colitis Organisation Congress, United European Gastroenterology Week, American College of Gastroenterology Annual Scientific Meeting) and clinical trial registration databases (ClinicalTrials.gov, EudraCT) to capture emerging Phase 2 and Phase 3 trial data. Articles published in any language were considered. In cases of duplicate publication, the most recent study meeting inclusion criteria was retained.

**Table S1. TL1A-targeted therapeutics in clinical and preclinical development for inflammatory bowel disease.**

| **Agent** | **Developer** | **Modality** | **Target(s)** | **Stage** | **Key Differentiator** |
| --- | --- | --- | --- | --- | --- |
| **First-generation monoclonal antibodies** | | | | | |
| **Tulisokibart (MK-7240)** | MSD | Humanised IgG1 mAb | TL1A | Phase 3 | Most advanced program; genetic CDx strategy (TNFSF15 rs1058532); IV induction / SC maintenance |
| **Afimkibart (RO7790121)** | Roche / Genentech | Fully human IgG1 mAb | TL1A | Phase 3 | Largest Phase 2b dataset (TUSCANY); treat-through design; biomarker subgroup secondary endpoints |
| **Duvakitug (TEV-48574)** | Teva / Sanofi | Human IgG1 mAb | TL1A | Phase 3 | SC-only regimen; highest Phase 2 remission rates (RELIEVE-UCCD); responder re-randomization design |
| **Long-acting / extended half-life monoclonal antibodies** | | | | | |
| **SPY002** | Spyre Therapeutics | Extended half-life IgG1 mAb | TL1A | Phase 2 | ~75-day half-life (3x first-gen); SKYLINE-UC platform trial evaluating monotherapy and combinations with anti-α4β7 (SPY001) and anti-IL-23 (SPY003); Q12–24W dosing potential |
| **XmAb942** | Xencor | Xtend Fc IgG1 mAb | TL1A | Phase 2b | >74-day half-life; class-leading potency; XENITH-UC Phase 2b enrolling; Q12W dosing |
| **BCD-261 (COMANDOR)** | Biocad | Anti-TL1A mAb | TL1A | Phase 2 | BCD-261-2/COMANDOR in moderate-to-severe Crohn's disease (planned n=510) |
| **BCD-261 (ULTRAMARINE)** | Biocad | Anti-TL1A mAb | TL1A | Phase 2 | BCD-261-4/ULTRAMARINE in moderate-to-severe ulcerative colitis (NCT07080034, planned n=495) |
| **ABS-101** | Absci | AI-designed mAb | TL1A | Phase 1 | First AI-designed anti-TL1A (de novo generative AI platform); Phase 1 dosing initiated May 2025; seeking development partner |
| **FG-M701** | AbbVie / FutureGen | Fully human mAb | TL1A | Preclinical | STEP platform; licensed Jun 2024 (US$150M upfront + up to US$1.56B milestones); extended dosing interval |
| **Bispecific antibodies** | | | | | |
| **RO7837195 (ex PF-07261271)** | Roche (ex-Pfizer) | p40 × TL1A bispecific | TL1A + IL-12/23 | Phase 2b | Most clinically advanced TL1A-containing bispecific; UC Phase 2b (NCT06979336); dual engagement of IL-12 and IL-23 pathways |
| **CLD-423** | Caldera Therapeutics | TL1A × IL-23p19 bispecific | TL1A + IL-23 | Phase 1 | First-in-class dual TL1A/IL-23 inhibition; Phase 1 first dosed Jan 2026; US$112.5M launch funding |
| **XmAb412** | Xencor | TL1A × IL-23p19 bispecific (Xtend Fc) | TL1A + IL-23 | Preclinical | Xtend Fc long-acting format; picomolar affinity for both targets; predicted human half-life ≥60 days; FIH planned 2026 |
| **HXN-1002** | Earendil Labs / Helixon Therapeutics | α4β7 × TL1A bispecific | TL1A + α4β7 | Preclinical | Enhanced α4β7 internalisation via TL1A co-engagement; licensed to Sanofi Apr 2025 |
| **HXN-1003** | Earendil Labs / Helixon Therapeutics | TL1A × IL-23p19 bispecific | TL1A + IL-23 | Preclinical | Dual neutralisation; superior efficacy in DSS colitis and IMQ psoriasis models versus IL-23 monotherapy |
| **LQ080** | Novamab | TL1A × IL-23p19 single-domain antibody | TL1A + IL-23 | Preclinical | Biparatopic single-domain antibody; long-acting subcutaneous; no immune complex formation |
| **SIM0709** | Simcere / Boehringer Ingelheim | TL1A × IL-23p19 bispecific | TL1A + IL-23 | Preclinical | Licensed Jan 2026 (up to €1.05B / US$1.26B); superior synergistic efficacy over combination in preclinical models |
| **ALX001** | Ailux Biotechnology | TL1A × IL-23 bispecific (2+2 format) | TL1A + IL-23 | Preclinical | Symmetric 2+2 format; high affinity (TL1A KD < 0.1 nM, IL-23 KD < 0.01 nM); selective DR3 blockade sparing DcR3 |
| **Oral TL1A modalities** | | | | | |
| **Oral anti-TL1A single-domain antibody** | Novamab | Oral nanobody | TL1A | Preclinical | Gut-restricted; TL1A–DR3 selective blockade sparing TL1A–DcR3; stable in simulated gastrointestinal fluid; active in murine DSS colitis |
| **DR3 receptor antagonist** | | | | | |
| **SL-325** | Shattuck Labs | Anti-DR3 monoclonal antibody | DR3 (receptor) | Phase 1 | Receptor-targeting approach (vs ligand); SAD/MAD in healthy volunteers (NCT07158437); FDA IND cleared Aug 2025 |

*Abbreviations: α4β7, alpha-4 beta-7 integrin; CDx, companion diagnostic; DR3, death receptor 3; DcR3, decoy receptor 3; DSS, dextran sulfate sodium; FDA, US Food and Drug Administration; Fc, fragment crystallisable region; FIH, first-in-human; IBD, inflammatory bowel disease; IgG, immunoglobulin G; IL, interleukin; IMQ, imiquimod; IND, investigational new drug; IV, intravenous; KD, equilibrium dissociation constant; mAb, monoclonal antibody; MAD, multiple ascending dose; SAD, single ascending dose; SC, subcutaneous; STEP, structure-based therapeutic engineering platform; TL1A, TNF-like ligand 1A; UC, ulcerative colitis.*
